# High-throughput assay for regulated secretion of neuropeptides in mouse and human neurons

**DOI:** 10.1016/j.jbc.2024.107321

**Published:** 2024-04-25

**Authors:** Urszula Baginska, Ganna Balagura, Ruud F. Toonen, Matthijs Verhage

**Affiliations:** 1 Department of Functional Genomics, Faculty of Exact Science, Center for Neurogenomics and Cognitive Research, VU University Amsterdam and VU Medical Center, Amsterdam, The Netherlands; 2 Department of Neurosciences, Rehabilitation, Ophthalmology, Genetics, and Maternal and Child Health, University of Genoa, Genoa, Italy; 3 Pediatric Neurology and Muscular Diseases Unit, IRCCS ‘G. Gaslini’ Institute, Genoa, Italy; 4 Human Genetics, Amsterdam University Medical Center, Amsterdam, The Netherlands

**Keywords:** neuropeptides, DCV exocytosis, Nanoluc, high-throughput assay

## Abstract

Neuropeptides are the largest group of chemical signals in the brain. More than 100 different neuropeptides modulate various brain functions and their dysregulation has been associated with neurological disorders. Neuropeptides are packed into dense core vesicles (DCVs), which fuse with the plasma membrane in a calcium-dependent manner. Here, we describe a novel high-throughput assay for DCV exocytosis using a chimera of Nanoluc luciferase and the DCV-cargo neuropeptide Y (NPY). The NPY-Nanoluc reporter colocalized with endogenous DCV markers in all neurons with little mislocalization to other cellular compartments. NPY-Nanoluc reported DCV exocytosis in both rodent and induced pluripotent stem cell-derived human neurons, with similar depolarization, Ca^2+^, RAB3, and STXBP1/MUNC18 dependence as low-throughput assays. Moreover, NPY-Nanoluc accurately reported modulation of DCV exocytosis by known modulators diacylglycerol analog and Ca^2+^ channel blocker and showed a higher assay sensitivity than a widely used single-cell low-throughput assay. Lastly, we showed that Nanoluc coupled to other secretory markers reports on constitutive secretion. In conclusion, the NPY-Nanoluc is a sensitive reporter of DCV exocytosis in mammalian neurons, suitable for pharmacological and genomic screening for DCV exocytosis genes and for mechanism-based treatments for central nervous system disorders.

Neuropeptides and neurotrophins are the largest and most diverse subgroup of chemical signals in the brain. Neuropeptide signaling is evolutionary conserved until some of the simplest life forms (1, 2, 3). In vertebrates, neuropeptides modulate brain circuits by the regulation of neuronal intrinsic properties and homeostasis and play crucial role in human body physiology and behavior (4, 5, 6). The importance of neuropeptide signaling is further underlined by their role in many brain disorders. In neurodegenerative disorders like Alzheimer’s or Parkinson’s disease, the expression of neuropeptides is altered (7, 8). Moreover, neuropeptides regulate neurotransmission and release of monoamines like dopamine and serotonin. Disruptions in monoamine pathways are hypothesized to be the underlying cause of many mental disorders like schizophrenia, depression, or bipolar disorders (9, 10). Therefore, the dysregulation of neuropeptides signaling is an important aspect during the characterization of neurodegenerative and mental disorders and the development of their treatments.

Neuropeptides accumulate in dense core vesicles (DCVs), often costored with classical neurotransmitters (11). In contrast to synaptic vesicles (SVs), DCVs do not use local reuptake/refill mechanisms. Instead, DCVs are filled with neuropeptide precursors at the Golgi from where they are transported to their release site (12, 13). Similarly, to the fusion of SVs, DCVs fuse in a calcium- and activity-dependent manner. However, the kinetics of DCV fusion are much slower than SVs, typically requiring more prolonged activity (14, 15, 16, 17). Neurons contain a large population of DCVs from which only ∼6% is released upon robust stimulation in mouse central nervous system neurons (18).

DCV exocytosis is commonly studied using optical probes consisting of the pH-sensitive EGFP variant super ecliptic pHluorin fused to DCV cargo such as neuropeptide Y (NPY) or brain-derived neurotrophic factor. This method detects single DCV fusion events during live-cell imaging with single-vesicle and high temporal resolution (18, 19, 20, 21, 22, 23). However, this method is less suitable as a high-throughput screening assay. Neuropeptide release can be quantified with method such as mass spectrometry or ELISAs that detect the release of endogenous neuropeptides with various levels of sensitivity. However, these methods typically require large cell samples, lack temporal resolution and are labor intensive (24).

Here, we exploit the luminescence-based Nanoluc system to establish a high-throughput assay for DCV exocytosis in neuronal cultures. The Nanoluc luciferase activity is not dependent on the presence of ATP, and the produced luminesce signal is characterized by intense brightness and long half-life (25). Nanoluc is widely used for the secretory assays (26, 27, 28). We fused Nanoluc to the canonical DCV cargo NPY. The NPY-Nanoluc reporter showed high colocalization with known DCV cargoes in both mouse and induced pluripotent stem cell (iPSC)-derived human neurons. The exocytosis of DCVs marked with NPY-Nanoluc was robustly detected after brief membrane depolarizations triggered with a high potassium solution (60 mM KCl). In addition, NPY-Nanoluc reported changes in basal exocytosis of DCVs and the total number of DCVs in cell lysates. The release of NPY-Nanoluc was successfully blocked by the L-type calcium channel blocker nimodipine (29, 30, 31) or the endoplasmic-reticulum (ER)-Golgi protein trafficking inhibitor brefeldin A (BFA) (32, 33) and enhanced by the diacylglycerol analog phorbol 12-myristate 13-acetate (PMA) (34, 35, 36). Genetic inactivation of STXBP1 or Rab3, essential genes for DCV exocytosis (19, 37) also lead to blockade of DCV exocytosis measured by NPY-Nanoluc. The power analysis revealed higher assay sensitivity of NPY-Nanoluc than low-throughput NPY-pHluorin assay. Lastly, we showed that Nanoluc coupled to other secretory markers reports on secretory pathways like constitutive release.

## Results

### NPY-Nanoluc reports DCV exocytosis in rodent central nervous system neurons

**Figure 1:**
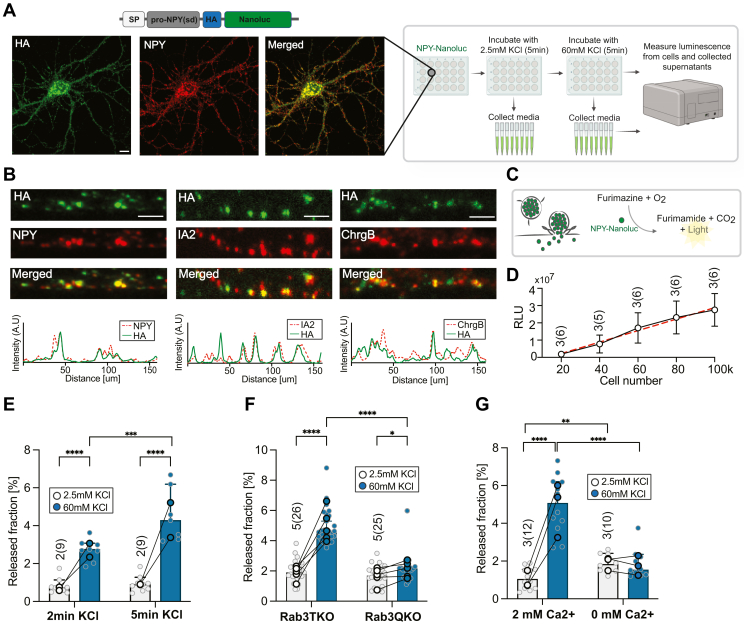
**NPY-Nanoluc reports DCV exocytosis in rodent central nervous system neurons.***A*, experimental design of the NPY-Nanoluc assay (created with BioRender.com) with a representative image of a neuron stained against HA-tag, NPY, and MAP2. *Above: graphical representation* of NPY-HA-Nanoluc construct. The scale bar represents 10um. *B*, colocalization of NPY-Nanoluc with endogenous DCVs cargoes IA-2 and chromogranin B (ChrgB) in hippocampal neurons. *Below:* line scan intensity plots for selected neurites. The scale bar represents 3 μm. *C*, *graphical representation* of NPY-Nanoluc release from fusing DCVs and enzymatic reaction upon the addition of furimazine substrate. *D*, linear correlation between cell number of hippocampal neurons expressing NPY-Nanoluc and luminescence intensity signal in relative light units (RLU). *E*, *bar plot* of released NPY-Nanoluc normalized to the luminescence signal from cell lysate (released fraction) upon stimulation for 2 or 5 min with 2.5 mM KCl or 60 mM KCl in hippocampal neurons. *F*, *bar plot* of released fraction upon 5 min stimulation of Rab3ABCD quadruple null mutant (Rab3QKO) compared to control Rab3BCD null (Rab3TKO) in hippocampal neurons. *G*, *bar plot* of released fraction upon 5 min stimulation of hippocampal neurons in 2 mM or 0 mM extracellular calcium. The concentration of calcium was adjusted for both evoked (60 mM KCl) and basal (2.5 mM KCl) release buffers. *Bar* represents median with 95% confidence interval, *single dot* represents measurement from single well, and the *highlighted, connected dots* represent median value for each independent experiment. Mann–Whitney or multiple Mann–Whitney was used. ∗*p* < 0.05, ∗∗*p* < 0.01, ∗∗∗*p* < 0.001, and ns *p* > 0.05. Table 1 shows results of statistical tests. DCV, dense core vesicle; HA-tag, YPYDVPDYA-tag; NPY, neuropeptide Y.

To detect DCV exocytosis using a luciferase assay in mammalian neurons, we fused Nanoluc (25) to the canonical DCV marker NPY. NPY is expressed in inhibitory neurons and its release dampens network activity (38, 39). However, when expressed also in other neurons, the signal sequence targets NPY constructs to the regulated secretory pathway with high specificity (18). To prevent that exogenous NPY affects the firing activity in neuronal cultures, we generated a truncated signaling dead variant of NPY in which the C-terminal peptide of NPY was removed and the amino acids Arg35 and Tyr36, essential for NPY receptor binding, were mutated to Alanines (40). Additionally, the construct was YPYDVPDYA-tagged for better detection with immunostaining (Fig. 1*A*). Lentiviral-mediated expression of NPY-Nanoluc 6 to 7 days prior to fixation showed robust colocalization with endogenous DCV markers at 18 days *in vitro* (DIV18) in primary mouse neurons (Fig. 1, *A* and *B*). To test the linear range of NPY-Nanoluc, we measured the luminescence signal (Fig. 1*C*, relative light units, RLU) from neuronal culture lysates plated at different densities (20,000–100,000 cells/well). The increase in cell density positively correlated with the linear increase in the RLU (R^2^ = 0.64, Fig. 1*D*). Taken together, these data indicate that NPY-Nanoluc is targeted to DCVs in primary neurons and the Nanoluc signal scales linearly with the number of neurons infected.

**Table 1:** Statistical analysis used to compare plotted values

Experiment	Condition	Statistical test used	*p* value
Figure 1*E*	2 min; basal *versus* evoked release	multiple Mann–Whitney	*p* < 0.0001
Figure 1*E*	5 min; basal *versus* evoked release	multiple Mann–Whitney	*p* < 0.0001
Figure 1*E*	evoked release; 2 min *versus* 5 min	Mann–Whitney	*p* = 0.0005
Figure 1*F*	RabTKO; basal *versus* evoked release	multiple Mann–Whitney	*p* < 0.000001
Figure 1*F*	RabQKO; basal *versus* evoked release	multiple Mann–Whitney	*p* = 0.017
Figure 1*F*	evoked release; RabTKO *versus* RabQKO	Mann–Whitney	*p* < 0.0001
Figure 1*G*	2 mM Ca2+; basal *versus* evoked release	multiple Mann–Whitney	*p* < 0.000001
Figure 1*G*	basal release; 0 mM Ca2+ *versus* 2 mM Ca2+	Mann–Whitney	*p* = 0.009
Figure 1*G*	evoked release; 0 mM Ca2+ *versus* 2 mM Ca2+	Mann–Whitney	*p* < 0.001
Figure 2*A*	evoked release; DMSO *versus* PMA	Mann*–*Whitney	*p* = 0.039
Figure 2*B*	evoked release; DMSO *versus* Nimodipine	Unpaired t test	*p* < 0.0001
Figure 2*C*	basal release; DMSO *versus* Dyngo-4a	Mann*–*Whitney	*p* = 0.0024
Figure 4*B*	evoked release; WT *versus* 0 mM Ca2+ 60 mM KCl	Kruskal*–*Wallis with Dunn’s post-hoc	*p* = 0.0007
Figure 4*B*	evoked release; WT *versus* STXBP1−/−	Kruskal*–*Wallis with Dunn’s post hoc	*p* = 0.0002
Figure 4*D*	basal release; DMSO *versus* PMA	Kruskal*–*Wallis with Dunn’s post hoc	*p* = 0.0004
Figure 4*E*	evoked release; DMSO *versus* 0 Ca2+	Kruskal*–*Wallis with Dunn’s post-hoc	*p* = 0.0043
Figure 4*G*	basal release; DMSO *versus* 2 h BFA	Kruskal*–*Wallis with Dunn’s post hoc	*p* = 0.0004
Figure 4*G*	basal release; DMSO *versus* 6 h BFA	KruskalvWallis with Dunn’s post hoc	*p* = 0.0001
Figure 4*G*	basal release; DMSO *versus* 10 h BFA	Kruskal*–*Wallis with Dunn’s post hoc	*p* < 0.0001
Figure 4*H*	evoked release; DMSO *versus* 2 h BFA	Kruskal*–*Wallis with Dunn’s post hoc	*p* = 0.0011
Figure 4*H*	evoked release; DMSO *versus* 6 h BFA	Kruskal*–*Wallis with Dunn’s post hoc	*p* < 0.0001
Figure 4*H*	evoked release; DMSO *versus* 10 h BFA	Kruskal*–*Wallis with Dunn’s post hoc	*p* < 0.0001
Figure 4*I*	RLU signal from cells; DMSO *versus* 6 h BFA	Kruskal*–*Wallis with Dunn’s post hoc	*p* = 0.0003
Figure 4*I*	RLU signal from cells; DMSO *versus* 6 h BFA	Kruskal*–*Wallis with Dunn’s post hoc	*p* < 0.0001
Figure 5*A*	2 h basal release; DMSO *versus* TTX	multiple Mann*–*Whitney	*p* < 0.0001
Figure 5*A*	6 h basal release; DMSO *versus* TTX	multiple Mann*–*Whitney	*p* < 0.0001
Figure 5*A*	24 h basal release; DMSO *versus* TTX	multiple Mann*–*Whitney	*p* < 0.0001
Figure 5*C*	24 h basal release; DMSO *versus* TTX	multiple Mann*–*Whitney	*p* = 0.001
Figure 5*B*	2 h RLU signal from cells; DMSO *versus* TTX	multiple Mann*–*Whitney	*p* = 0.003
Figure 5*B*	6 h RLU signal from cells; DMSO *versus* TTX	multiple Mann*–*Whitney	*p* < 0.0001
Figure 5*B*	24 h RLU signal from cells; DMSO *versus* TTX	multiple Mann*–*Whitney	*p* < 0.0001
Figure 5*D*	24 h RLU signal from cells; DMSO *versus* TTX	Two-way ANOVA	*p* = 0.01
Fig. S1*C*	evoked/basal ratio; RabTKO *versus* RabQKO	Mann–Whitney	*p* < 0.0001
Fig. S1*E*	evoked/basal ratio; 0 Mm Ca2+ *versus* 2 mM Ca2+	Mann–Whitney	*p* < 0.0001
Fig. S2*A*	0 mM Ca2+; basal *versus* evoked release	multiple Mann–Whitney	*p* = 0.03
Fig. S2*A*	0.1 mM; 1 mM; 2 mM; 5 mM; basal *versus* evoked release	multiple Mann–Whitney	*p* < 0.000001
Fig. S2*B*	evoked/basal release; 0 mM *versus* 0.1 mM	Kruskal*–*Wallis with Dunn’s post hoc	*p* = 0.0546
Fig. S2*B*	evoked/basal release; 0 mM *versus* 1 mM	Kruskal*–*Wallis with Dunn’s post hoc	*p* < 0.0001
Fig. S2*B*	evoked/basal release; 0 mM *versus* 2 mM	Kruskal*–*Wallis with Dunn’s post hoc	*p* < 0.0001
Fig. S2*B*	evoked/basal release; 0 mM *versus* 5 mM	Kruskal*–*Wallis with Dunn’s post hoc	*p* = 0.0091
Fig. S3*A*	evoked/basal release; DMSO *versus* PMA	Unpaired t test	*p* = 0.012
Fig. S3*C*	evoked/basal release; DMSO *versus* Nimodipine	Mann*–*Whitney	*p* = 0.0003
Fig. S3*E*	evoked/basal release; DMSO *versus* Dyngo-4a	Mann*–*Whitney	*p* = 0.011
Fig. S4*B*	evoked/basal release; WT *versus* 0 Ca2+ 60 mM KCl	Kruskal*–*Wallis with Dunn’s post hoc	*p* = 0.0001
Fig. S4*B*	evoked/basal release; WT *versus* STXBP1−/−	Kruskal*–*Wallis with Dunn’s post hoc	*p* = 0.0034
Fig. S4*D*	evoked/basal release; DMSO *versus* 0 mM Ca2+	Kruskal*–*Wallis with Dunn’s post hoc	*p* < 0.0001
Fig. S4*D*	evoked/basal release; DMSO *versus* PMA	Kruskal*–*Wallis with Dunn’s post hoc	*p* = 0.016
Fig. S4*E*	evoked/basal release; DMSO *versus* 6 h BFA	Kruskal*–*Wallis with Dunn’s post hoc	*p* = 0.0068
Fig. S4*E*	evoked/basal release; DMSO *versus* 10 h BFA	Kruskal*–*Wallis with Dunn’s post hoc	*p* = 0.0007
Fig. S5*B*	NPY; 2 h basal release; DMSO *versus* TTX	multiple Mann–Whitney	*p* = 0.000003
Fig. S5*B*	NPY; 6 h basal release; DMSO *versus* TTX	multiple Mann–Whitney	*p* = 0.0022
Fig. S5*B*	NPY; 24 h basal release; DMSO *versus* TTX	multiple Mann–Whitney	*p* = 0.0022
Fig. S5*C*	NPY; 6 h RLU signal from cells; DMSO *versus* TTX	multiple Mann–Whitney	*p* = 0.0022
Fig. S5*C*	NPY; 24 h RLU signal from cells; DMSO *versus* TTX	multiple Mann–Whitney	*p* = 0.0043
Fig. S6*A*	basal release; DMSO *versus* AP5/DNQX	Mann*–*Whitney	*p* = 0.0209
Fig. S6*D*	2 h basal release; DMSO *versus* AP5/DNQX	One-way ANOVA	*p* ≤ 0.0001
Fig. S6*D*	2 h basal release; DMSO *versus* TTX	One-way ANOVA	*p* = 0.0002

BFA, brefeldin A; DMSO, dimethyl sulfoxide; NPY, neuropeptide Y; RLU, relative light units; TTX, tetrodotoxin.

To assess if NPY-Nanoluc reporter detects evoked DCV exocytosis, mouse hippocampal neurons were plated at 60,000 cells/well in 24-well plates. To measure basal (driven by spontaneous network activity) and evoked DCV exocytosis (driven by membrane depolarization), wells were first incubated with normal Tyrode’s imaging solution containing 2.5 mM KCl (basal release from DCVs) and subsequently with a Tyrode’s solution containing 60 mM KCl (evoked release from DCVs). The media from each treatment were collected and RLU activity was measured. The released fraction was calculated as a percentage of released NPY-Nanoluc to the activity measured from the corresponding cell lysates (total DCV pool, Fig. 1, *A* and *C*). We first compared basal and evoked release of NPY-Nanoluc during either a 2-min or 5-min incubation time. For both incubation times, a significant increase in NPY-Nanoluc release upon stimulation was observed compared to basal release with released fractions of 2.8% and 4.3%, respectively, for the 2- and 5-min stimulation. Released fractions measured during the 2- or 5-min incubation with normal Tyrode’s were 0.7% and 0.9% (Fig. 1*E*). Hence, NPY-Nanoluc is released from DCVs upon depolarization and the released fraction is comparable to low-throughput assays and electrical stimulation (18). Because of the slightly higher effect sizes during the 5 min stimulations, we chose this timeframe for all future experiments.

To test for the specificity of NPY-Nanoluc as a DCV exocytosis reporter, we used hippocampal neurons lacking all four Rab3 isoforms (RAB3ABCD) (41). Knock out of Rab3ABCD in mouse hippocampal neurons reduces DCV fusion by more than 90% (18), whereas SV fusion remains largely intact (28). The release of NPY-Nanoluc in control Rab3BCD (RabTKO) null mutant neurons showed a typical increase upon stimulation (4.6% of the total pool) compared to basal release from DCVs (1.9% of the total pool). In contrast, evoked DCV fusion in Rab3ABCD (RabQKO) null mutant neurons was strongly impaired (2.2%) and comparable to the release fraction measured without stimulation (1.7%, Fig. 1*F*). Because the basal and evoked NPY-Nanoluc release is measured within the same well, the ratio of evoked/basal DCV exocytosis can be determined for each well. The ratios of evoked to basal release from DCVs of RabTKO and RabQKO showed significant differences (RabTKO:2.37, RabQKO:1.3, Fig. S1*C*). Hence, NPY-Nanoluc exocytosis is largely Rab3A-dependent.

DCV exocytosis is calcium-dependent and removal of extracellular Ca^2+^ abolishes DCV exocytosis (42, 43). As expected, 60 mM KCl stimulation in 0 mM extracellular Ca^2+^ did not trigger NPY-Nanoluc release (1.5%) compared to an evoked released fraction of 5.1% in 2 mM extracellular calcium. Interestingly, basal DCV exocytosis measured in 0 mM extracellular calcium (1.8%) was significantly higher as compared to basal DCV exocytosis in 2 mM calcium (1%, Fig. 1*G*). Additionally, we measured basal and evoked DCV fusion in mouse cortical neuron cultures at different extracellular calcium concentrations (0–5 mM), which showed that the increasing extracellular calcium increases DCV exocytosis, which reaches plateau at 1 mM (Fig. S2).

Overall, these results show that NPY-Nanoluc correctly targets to DCVs and reports on regulated exocytosis of these vesicles.

### NPY-Nanoluc reports DCV exocytosis upon treatment with network activity modulators

**Figure 2:**
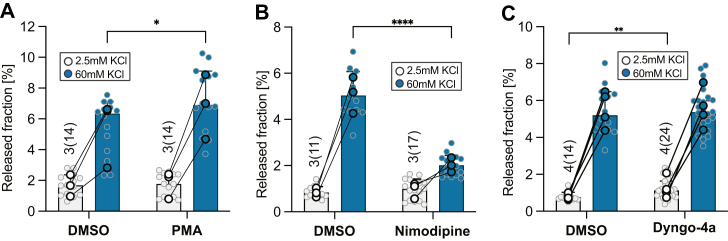
**NPY-Nanoluc reports DCV exocytosis upon treatment with network activity modulators.***A*, *bar plot* of released fraction upon 5 min stimulations of hippocampal neurons treated with PMA (1 μM) or DMSO. PMA was added only to the evoked stimulation buffer (60 mM KCl). *B*, *bar plot* of released fraction of cortical neurons treated with nimodipine (30 μM) or DMSO. Nimodipine was added to both basal stimulation buffer (2.5 mM KCl) and evoked stimulation buffer (60 mM KCl). *C*, *bar plot* of released fraction of cortical neurons treated with Dyngo-4a (10 μM) or DMSO. Dyngo-4a was added to both basal stimulation buffer (2.5 mM KCl) and evoked stimulation buffer (60 mM KCl). For nonnormally distributed data, *bar* represents median value with 95% confidence interval; *single dot* represents measurement from single well; and the *highlighted, connected dots* represent median value for each independent experiment. Mann–Whitney was used. ∗*p* < 0.05, ∗∗*p* < 0.01, ∗∗∗*p* < 0.001, and ns *p* > 0.05. For normally distributed data, *bar* represents mean value with SD and the *highlighted, connected dots* represent mean value for each independent experiment. Unpaired *t* test was used, ∗*p* < 0.05, ∗∗*p* < 0.01, ∗∗∗*p* < 0.001, and ns *p* > 0.05. Table 1 shows results of statistical tests. DCV, dense core vesicle; DMSO, dimethyl sulfoxide; NPY, neuropeptide Y; PMA, phorbol 12-myristate 13-acetate.

Next, we tested the ability of the NPY-Nanoluc assay to detect effects of known modulators of DCV exocytosis. First, we tested the diacylglycerol analog PMA, known to increase priming and release of secretory granules in PC12 and chromaffin cells (34, 35, 36). The addition of 1 μM PMA produced a small but significant increase in evoked release from DCVs (released fraction in dimethyl sulfoxide (DMSO) and PMA was 6.3% and 6.9%, respectively, Fig. 2*A*), which was also apparent when the ratio of evoked/basal DCV fusion was calculated (Fig. S3*A*). Second, neuropeptide release from primary neuronal cultures depends on L-type Ca^2+^ channels (29, 30, 31). Application of the L-type Ca^2+^ channel antagonist nimodipine (30 μM) did not change basal NPY-Nanoluc release but evoked NPY-Nanoluc release was strongly reduced (2.0% *versus* 5.0% of the pool, Fig. 2*B*). Lastly, we tested the effect of the dynamin inhibitor Dyngo-4a (27, 28), which inhibits DCV exocytosis in single isolated mouse neurons (37). Interestingly, the treatment of Dyngo-4a did not affect evoked DCV exocytosis (DMSO: 5.2%, Dyngo-4a: 5.4%, Fig. 2*C*) but significantly increased the release of NPY-Nanoluc during basal release (DMSO: 0.8%, Dyngo-4a: 1.1%, Fig. 2*C*).

Overall these data show that the NPY-Nanoluc assay detects changes in DCV exocytosis upon treatment of known network activity modulators.

### NPY-Nanoluc has higher assay sensitivity than low-throughput assay for DCV exocytosis

In order to predict the sensitivity and statistical power of the Nanoluc assay, we performed power analysis for each of the conditions tested in Figures 1 and 2, and Fig. S2 for previously published data (Persoon *et al.*, 2019) comparing DCV released fraction with single-cell NPY-pHluorin. The number of observations per group needed to reach 80% of statistical power was predicted *in silico* based on the effect size calculated for each condition (only conditions with effect size >0.03 are shown). Both evoked DCV fusion assays showed positive correlation between the mean differences (the percentage difference between average DCV released fraction of WT and tested condition) and the number of observations per group needed to 80% power (evoked NPY-Nanoluc: R^2^ =0.96, NPY-pHluorin: R^2^ = 0.99). However, the evoked NPY-Nanoluc showed much lower number of observations per group needed to obtained 80% power than NPY-pHluorin; conditions with 60% difference in mean evoked DCV fusion reached 80% of predicted power with only 2 to 3 observations per group for NPY-Nanoluc, whereas the same 60% mean difference measured with NPY-pHluorin assay required 47 observations. The correlation between mean difference and the number of observations needed to reach 80% for basal NPY-Nanoluc release showed lower correlation (R^2^ = 0.63). Nevertheless, the number of observations per group needed to reach 80% power for basal NPY-Nanoluc release with mean difference of 58% was lower than the mean difference of 60% obtained with NPY-pHluorin (basal NPY-Nanoluc: 23, NPY-pHluorin: 47, Figure 3*A* and Table 2).

**Figure 3:**
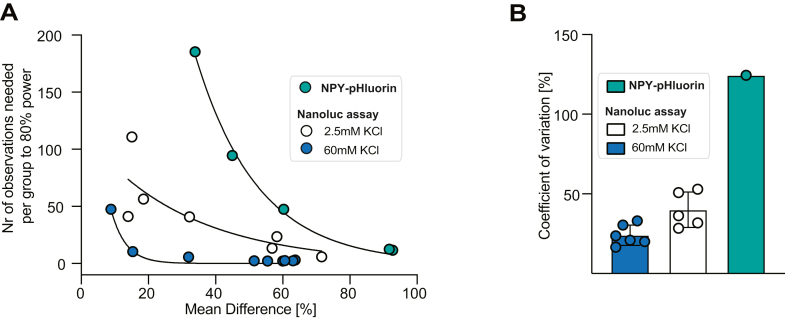
**NPY-Nanoluc has higher assay sensitivity than low-throughput assay for DCV exocytosis.***A*, the relationship between mean differences [%] and the number of observations per group needed to reach 80% of statistical power for every condition measured with NPY-Nanoluc (Figure 1, Figure 2, and S2) or single-cell NPY-pHluorin assay (data previously published in Persoon *et al.*, 2019). *B*, the coefficient of variation for DCV exocytosis in WT neurons measured with NPY-pHluorin (data previously published in Persoon *et al.*, 2019) or NPY-Nanoluc (Figure 1, Figure 2, and S2). DCV, dense core vesicle; NPY, neuropeptide Y.

**Table 2:** Results of statistical power analysis

Source of data	Conditions (WT *versus* challenged condition)	Effect size	Number of observations per group needed to 80% power	Mean differences [%]	Coefficient of variation for WT	Coefficient of variation for challenged condition
Persoon *et al.*, 2019	Rab3A+/+BCD−/− *versus* Rab3ABCD−/−	1,22	11,55	92,64	124,40	162,52
Persoon *et al.*, 2019	Rab3A+/+BCD−/− *versus* Rab3ABCD−/− + Rab3A	0,29	185,31	33,88	124,40	196,75
Persoon *et al.*, 2019	Rab3A+/+BCD−/− *versus* Rab3ABCD−/− + Rab3B	1,17	12,46	91,62	124,40	254,61
Persoon *et al.*, 2019	Rab3A+/+BCD−/− *versus* Rab3ABCD−/− + Rab3C	0,41	94,6	44,96	124,40	217,92
Persoon *et al.*, 2019	Rab3A+/+BCD−/− *versus* Rab3ABCD−/− + Rab3D	0,58	47,50	60,23	124,40	254,01
Figure 1*F*	basal release; RabTKO *versus* RabQKO	0,62	41,26	13,95	36,37	39,34
Figure 1*G*	basal release; 2 mM Ca2+ *versus* 0 mM Ca2+	1,82	5,90	71,52	50,76	25,39
Fig. S2*A*	Basal release; 2 mM Ca2+ *versus* 0.1 mM Ca2+	0,38	110,87	15,08	52,95	64,57
Fig. S2*A*	basal release; 2 mM Ca2+ *versus* 5 mM Ca2+	0,63	40,76	32,23	52,95	60,38
Fig. S2*A*	basal release; 2 mM Ca2+ *versus* 0 mM Ca2+	0,84	23,47	58,22	52,95	72,82
Figure 2*B*	basal release; DMSO *versus* Nimodipine	0,53	56,36	18,54	31,85	44,35
Figure 2*C*	basal release; DMSO *versus* Dyngo-4a	1,13	13,37	56,76	28,42	43,93
Figure 1*E*	evoked release; 2 min *versus* 5 min	3,04	3,03	63,69	20,01	28,61
Figure 1*F*	evoked release; RabTKO *versus* RabQKO	4,66	2,20	55,49	23,64	41,14
Figure 1*G*	evoked release; 2 mM Ca2+ *versus* 0 mM Ca2+	4,40	2,27	63,02	30,39	42,53
Fig. S2*A*	evoked release; 2 mM Ca2+ *versus* 0 mM Ca2+	3,73	2,54	60,60	16,55	56,15
Fig. S2*A*	evoked release; 2 mM Ca2+ *versus* 0.1 mM Ca2+	4,44	2,26	51,50	16,55	33,16
Fig. S2*A*	evoked release; 2 mM Ca2+ *versus* 5 mM Ca2+	1,30	10,31	15,36	16,55	15,24
Fig. S2*A*	evoked release; 2 mM Ca2+ *versus* 1 mM Ca2+	0,58	47,50	8,84	16,55	23,95
Figure 2*A*	evoked release; DMSO *versus* PMA	1,85	5,72	31,98	33,04	29,08
Figure 2*B*	evoked release; DMSO *versus* nimodipine	5,20	2,08	60,03	20,94	21,73

DMSO, dimethyl sulfoxide; PMA, phorbol 12-myristate 13-acetate.

Moreover, the coefficient of variation for DCV release in WT condition measured with NPY-pHluorin was 124%, which agrees with previous reports showing high coefficient of variation for NPY-pHluorin assay (23). The coefficient of variation for evoked and basal NPY-Nanoluc release was 16.6 to 33% and 28.4 to 53%, respectively (Fig. 3*B* and Table 2).

Therefore, NPY-Nanoluc assay shows higher assay sensitivity and lower variation than low-throughput pHluorin based assay.

### NPY-Nanoluc as a high-throughput assay to study DCV exocytosis in iPSC-derived human neurons

**Figure 4:**
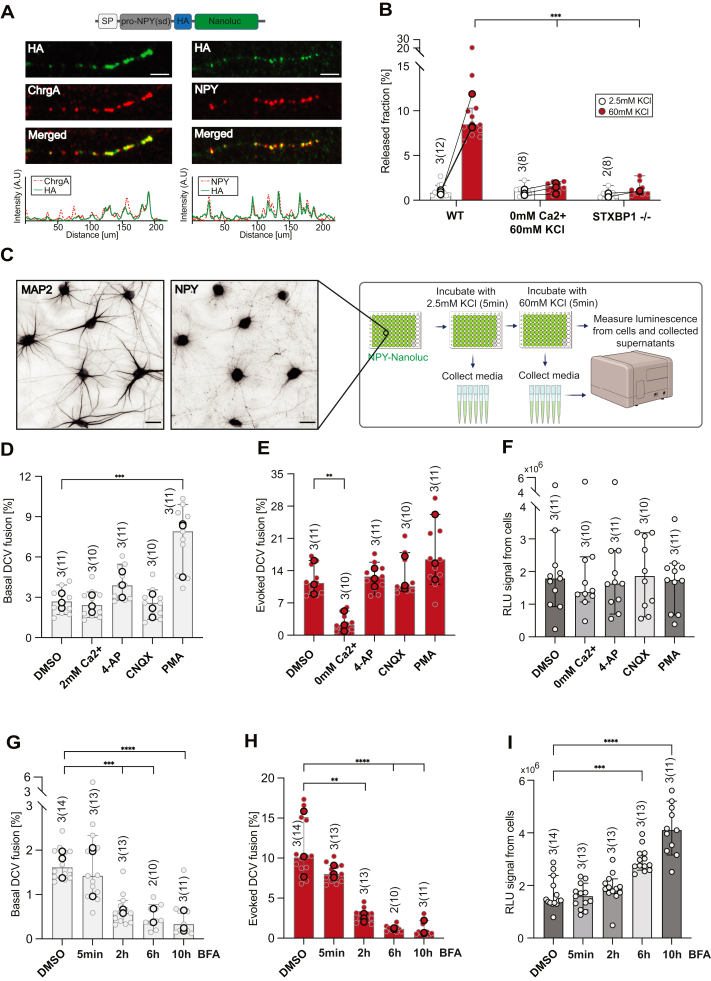
**NPY-Nanoluc as a high-throughput assay to study DCV exocytosis in iPSC-derived human neurons.***A*, colocalization of NPY-Nanoluc with DCVs cargoes in iNeurons (DIV21-25) in 24-well plates. *Below:* the *intensity plots* for selected neurites. The scale bar represents 3 μm. *B*, *bar plot* of released fraction upon 5 min stimulations in 0 mM extracellular calcium (the concentration of calcium was adjusted for evoked release buffer) and homozygous STXBP1 null mutant in iNeurons at DIV14 in 24-well plates without glia. *C*, experimental design of the NPY-Nanoluc assay in 96-well plates (created with BioRender.com) with the representative image of typical example of iNeurons cultured on glia layer in 96-well plates stained against NPY and MAP2. The scale bar represents 30 μm. All experiments described in (*D*–*I*) were carried in 96-well plates with glia. *D*, *bar plot* of basal release (2.5 mM KCl) of NPY-Nanoluc in iNeurons (DIV21-25) upon treatment with 4-AP (100 nM), CNQX (10 μM), PMA (1 μM), or DMSO. All three compounds were added to washing buffer, basal, and evoked release buffers. As a control for evoked release in 0 Ca2+ condition, neurons were treated with normal basal release buffer (2 mM Ca2+, 2.5 mM KCl). *E*, *bar plot* of evoked release (60 mM KCl) of NPY-Nanoluc for the conditions presented in (*D*). *F*, RLU signal from cells lysates for the conditions presented in (*D* and *E*). *G*, *bar plot* of basal release (2.5 mM KCl) of NPY-Nanoluc in iNeurons (DIV21-25) upon treatment with BFA (5 μM) for either 5 min, 2 h, 6 h, or 10 h. BFA was added to washing buffer, basal, and evoked release buffers. *H*, *bar plot* of evoked release (60 mM KCl) of NPY-Nanoluc for the conditions presented in (G). BFA was added to both basal and evoked release buffers in (*G* and *H*). *I*, RLU signal from cells lysates for the conditions presented in (*G* and *H*). *Bar* represents median value with 95% confidence interval; *single dot* represents measurement from single well; and the *highlighted dots* represent median value for each independent experiment. Kruskal–Wallis with Dunn’s post hoc comparison was used. ∗*p* < 0.05, ∗∗*p* < 0.01, ∗∗∗*p* < 0.001, and ns *p* > 0.05. Table 1 shows results of statistical tests. BFA, brefeldin A; CNQX, α-amino-3-hydroxy-5-methyl-4-isoxazolepropionic acid; DCV, dense core vesicle; DIV, days *in vitro*; DMSO, dimethyl sulfoxide; NPY, neuropeptide Y; PMA, phorbol 12-myristate 13-acetate; RLU, relative light units.

We next tested if NPY-Nanoluc assay can be used to study DCV exocytosis in human iPSC-derived neurons (iNeurons) (44, 45, 46, 47). iNeurons expressing NPY-Nanoluc, through viral infection 6 to 7 days prior to the experiment, showed high colocalization of NPY-Nanoluc (visualized with HA-antibodies) with known DCV marker chromogranin A (Fig. 4*A*). Like in rodent neurons, DCV exocytosis was induced by depolarization and blocked in 0 mM extracellular calcium (WT 8.5%, 0 mM Ca2+:1.6%, Fig. 4*B*). STXBP1/MUNC18 is essential for DCV exocytosis in mouse neurons (37). We generated NGN2-inducible iPSC cell line with homozygous inactivation of the STXBP1 gene using CRISPR-Cas9 (Fig. S4*A*). In iNeurons lacking STXBP1, exocytosis of DCVs labeled with NPY-Nanoluc during evoked release was drastically decreased; 8.5% and 1.0% of the total DCV pool for WT and STXBP1−/−, respectively. The released fraction during basal NPY-Nanoluc release also showed a trend toward decreased exocytosis (not significantly, WT: 0.8% and STXBP1−/−: 0.5% of the total DCV pool, Fig. 4*B*). These data show that the NPY-Nanoluc assay can also be used to study DCV exocytosis in iPSC-derived human neurons.

To increase the assay's throughput, we cultured iNeurons in 96-well plates and tested the effect of network activity modulating compounds. The luminescence assay was performed at DIV21 to 25 on iNeurons infected with lentiviral particles expressing NPY-Nanoluc 6 to 7 days prior to the experiment (Fig. 4*C*). The effect of three compounds on basal and evoked NPY-Nanoluc release was tested: the potassium channel blocker 4-Aminopyridine (100 μM), which increases network activity (48, 49), the α-amino-3-hydroxy-5-methyl-4-isoxazolepropionic acid receptor blocker cyanquixaline (10 μM), and PMA (1 μM), which increased DCV exocytosis in mouse neurons (Fig. 2*A*). PMA treatment significantly increased basal release from DCVs (7.9% of the total pool) compared to 2.7% in DMSO control condition. Basal DCV fusion upon 4-AP treatment showed a trend toward increase to 3.9%, while the treatment of α-amino-3-hydroxy-5-methyl-4-isoxazolepropionic acid did not affect basal NPY-Nanoluc release (Fig. 4*D*). As expected, 0 mM extracellular calcium blocked evoked DCV exocytosis (2.2% of the total pool). The exocytosis of DCV during evoked release showed no significant differences for all three tested compounds (Fig. 4*E*).

Finally, we tested if NPY-Nanoluc can be used to detect changes in DCV cargo content of the neurons using BFA. BFA inhibits the transport between the ER and Golgi, which leads to the accumulation of the newly produced protein in the ER/Golgi and blocks the production of Golgi-derived vesicles (32, 33). Preincubation of iNeurons with BFA (5 μM) for different time periods (5 min, 2 h, 6 h, and 10 h) significantly decreased basal DCV exocytosis already after 2 h incubation (DMSO: 1.6%, 2 h BFA: 0.6%) and it dropped further to 0.4%, and 0.3%, after 6 h, and 10 h BFA treatment, respectively. A similar magnitude of reduction was observed for evoked NPY-Nanoluc release, which dropped from 10.1%, to 3.0%, 1.1%, and 0.8% of the total DCV pool for DMSO, 2 h BFA, 6 h BFA, and 10 h BFA, respectively. Treatment of cultures with BFA for 5 min did not affect basal or evoked DCV fusion (Fig. 4, *G* and *H*). BFA treatment for 6 and 10 h led to a significant increase of NPY-Nanoluc signal measured from the cell lysates (DMSO: 1.4 × 10^6^, 6 h BFA: 2.8 × 10^6^, 10 h BFA: 4.1 × 10^6^) (Fig. 4*I*).

Hence, blocking Golgi-derived vesicle trafficking with BFA had a remarkably fast and robust effect on both NPY-Nanoluc release and intracellular accumulation.

### Nanoluc assay for the detection of other secretory pathways in iNeurons

Finally, we tested the Nanoluc-based assay for the analysis of the constitutive secretory pathway in iNeurons using the previously described secreted Nanoluc (sec-Nanoluc) consisting of the interleukin-6 secretory signal fused to Nanoluc (50). In contrast to DCV exocytosis, release of vesicles from the constitutive pathway is expected not to be affected by blocking neuronal culture activity (51, 52). Moreover, tetrodotoxin (TTX) treatment leads to the accumulation of DCVs at rat hippocampal presynaptic boutons and motor neurons terminals (53, 54). To test this and confirm correct targeting of sec-Nanoluc to the constitutive pathways, we compared the effect of the sodium channel blocker TTX (1 μM), which prevents action potential generation and hence network activity, on the release of NPY-Nanoluc with sec-Nanoluc.

**Figure 5:**
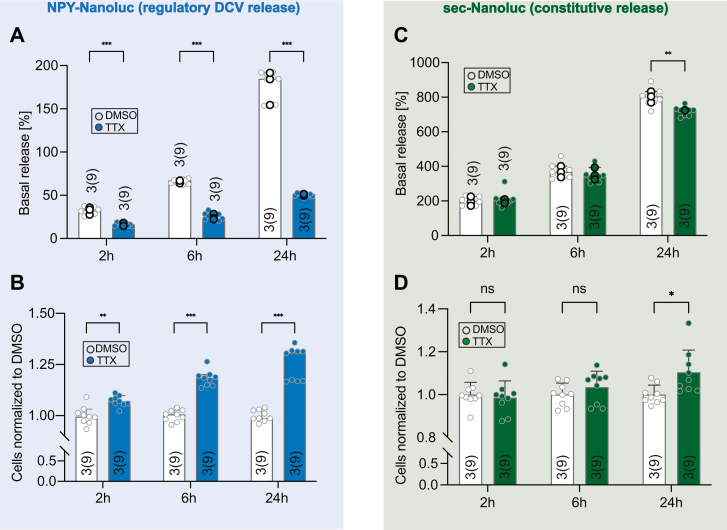
**Nanoluc assay for the detection of other secretory pathways in iNeurons.***A*, *bar plot* of released fraction of NPY-Nanoluc in culture media over different incubation time (2 h, 6 h, 24 h) with either DMSO or TTX (1 μM) in iNeurons (DIV21-25). *B*, RLU signal from cells lysates expressing NPY-Nanoluc for the conditions presented in (*A*), normalized to the DMSO control. *C*, *bar plot* of released fraction of sec-Nanoluc in culture media over different incubation time (2 h, 6 h, 24 h) with either DMSO or TTX (1 μM) in iNeurons (DIV21-25). *D*, RLU signal from cells lysates expressing sec-Nanoluc for the conditions presented in (*C*), normalized to the DMSO control. For nonnormally distributed data, *bar* represents median value with 95% confidence interval; *single dot* represent measurement from single well; and the *highlighted dots* represent median value for each independent experiment. Multiple Mann–Whitney was used. ∗*p* < 0.05, ∗∗*p* < 0.01, ∗∗∗*p* < 0.001, and ns *p* > 0.05. For normally distributed data, *bar* represents mean value with SD; *single dot* represents measurement from single well; and the *highlighted dots* represent mean value for each independent experiment. Two-way ANOVA was used, ∗*p* < 0.05, ∗∗*p* < 0.01, ∗∗∗*p* < 0.001, and ns *p* > 0.05. Table 1 shows results of statistical tests. DIV, days *in vitro*; DMSO, dimethyl sulfoxide; NPY, neuropeptide Y; RLU, relative light units; TTX, tetrodotoxin.

iPSC lines stably expressing either NPY-Nanoluc or sec-Nanoluc *via* lentiviral infection were generated. iNeurons cultured in 96-well plates were treated with TTX or DMSO for 2 h, 6 h, and 24 h and the release of both secretory markers was evaluated by measuring the luminescence signal from the media collected after the different incubation periods. The luminescence signal of NPY-Nanoluc in the media from control cultures increased with the incubation time (33.5%, 67.1%, and 167.6% for 2 h, 6 h, and 24 h, respectively). As expected, the released fraction of NPY-Nanoluc upon TTX treatment was drastically decreased for all of the tested incubation periods: 16.3%, 23.6%, and 51.3% for 2 h, 6 h, and 24 h incubation time, respectively (Fig. 5*A*). TTX treatment increased the NPY-Nanoluc signal in cell lysates already after 2 h incubation (∼7%) and 6 h and 24 h increased the signal further to ∼18% and ∼31%, respectively (Fig. 5*B*). Compared to NPY-Nanoluc, sec-Nanoluc showed much higher released fraction in control conditions (2 h: 199.7%, 6 h: 366.1%, 24 h: 804.8%), but TTX treatment did not affect this until 6 h incubation (2 h: 203.9% and 6 h: 340.1%). A small decrease of the released fraction of sec-Nanoluc was detected after 24 h incubation with TTX (725.5%, Fig. 5*C*). TTX treatment did not affect the sec-Nanoluc luminescence signal from cells lysates, but 24 h TTX incubation lead to an approximate 10% increase (Fig. 5*D*).

It has been shown that even healthy cultures experience cell rupture/leakage (55), which in our assay might add nonspecific signal to our NPY-Nanoluc detection in the medium. To control for the release of Nanoluc from cell rupture/leakage, we overexpressed either NPY-Nanoluc or Nanoluc with nuclear localization signal (NLS-Nanoluc) by lentiviral-mediated infection 7 days before the experiment in iNeurons. The release of NPY-Nanoluc over 2 h, 6 h, or 24 h incubation time with or without TTX showed the same phenotype as shown in Figure 5, *A* and *B*. The release of NLS-Nanoluc, however, was dramatically lower than the release of NPY-Nanoluc; for all incubation time points the release of NLS-Nanoluc was between 0.5 to 1.1% from the total pool (Fig. S5*B*).

These results show that the Nanoluc assay in neurons can be used for detecting changes in the exocytosis of both regulatory and constitutive pathway with little contribution from cell leakage/death.

## Discussion

We describe a luciferase-based high-throughput assay for the detection of basal and evoked DCV exocytosis in two *in vitro* models: primary mouse and human iPSC-derived neuronal culture. NPY-Nanoluc showed colocalization with known DCV markers (Figs. 1*B* and 4*A*). Multiple experiments indicated that NPY-Nanoluc release reports on DCV exocytosis: the release of NPY-Nanoluc was blocked upon removal of extracellular calcium (Fig. 1*G*) or knock out of genes involved in DCV exocytosis, Rab3a (Fig. 1*F*) and STXBP1 (Fig. 4*B*). Moreover, the L-type channel blocker nimodipine drastically decreased and PMA increased NPY-Nanoluc release (Figs. 2, *A* and *B*, 4*D*). The treatment with BFA or TTX showed that NPY-Nanoluc assay can also be used to study DCV accumulation in neurons (Figs. 4*I* and 5*B*). Power analyses revealed that NPY-Nanoluc has a higher assay sensitivity than the low-throughput NPY-pHluorin assay (Fig. 3). Lastly, we showed that in iPSC-derived human neurons the release of cargo through the constitutive pathway can be detected by using sec-Nanoluc, which is action potential independent (Fig. 5).

Here, we show that mouse hippocampal neurons upon stimulation for 5 min with high potassium release 4 to 6% of NPY-Nanoluc (Figs. 1, *E* and *G* and 2*A*). Previous data from hippocampal autaptic culture showed that 24 s of electrical field stimulation at 50 Hz leads to the fusion of 6% of DCVs visualized with NPY-pHluorin and by the end of the stimulation the releasable pool of the DCVs is depleted (18, 23). Hence, the two assays report a similar fraction of evoked DCV cargo release. In single-cell assays like the NPY-pHluorin assay, the assessment of basal fusion events is very hard, given the rarity of these events. In contrast, the NPY-Nanoluc assay can be used to assess (changes in) basal DCV exocytosis already after 5 min incubation. The treatment of hippocampal neurons with ionotropic glutamate receptors blockers (AP5/DNQX) significantly decreased the basal release of NPY-Nanoluc during 5 min incubation (Fig. S6*A*). The basal released fraction of NPY-Nanoluc for iNeurons at DIV14 was 0.83% (Fig. 4*B*), whereas the released fraction for iNeurons at DIV21 to 25 was 1.6 to 2.7% (Fig. 4, *D* and *G*). For human NGN2-induced neurons, the number of neurons showing basal excitatory postsynaptic currents increases drastically from DIV14 to DIV21 (44). Hence, the observed increase of NPY-Nanoluc basal release between DIV14 and DIV21 to 25 may be caused by this concomitant increase in network activity. Indeed, the incubation of iNeurons at DIV21 to 25 with TTX confirmed that the basal release of NPY-Nanoluc is action potential-dependent (Fig. 5*A*).

DCV exocytosis in neurons lacking Rab3 is abolished (19). Here, we show that the evoked release of NPY-Nanoluc was also blocked in neurons lacking Rab3 (Fig. 1*F*). Interestingly, in hippocampal neurons lacking Rab3, the basal release of NPY-Nanoluc was not affected (Fig. 1*F*). It seems safe to assume that a fraction of NPY-Nanoluc released from neurons without K^+^ depolarization is in fact also “evoked” release due to spontaneous activity known to exist in cultured neurons. This assumption is supported by the fact that TTX reduces basal NPY-Nanoluc release in both mouse hippocampal neurons (Fig. S6*D*) and iPSC-derived iNeurons (Fig. 5*A*). Furthermore, basal NPY-Nanoluc release from STXBP1 KO iNeurons was lower than WT (WT: 0.8% and STXBP1−/−: 0.5%, Fig. 4*B*), suggesting that at least approximately half the basal NPY-Nanoluc release is soluble NSF attachment protein receptor-dependent (and originates from DCVs). The other half of basal NPY-Nanoluc release might be due to cell rupture during washing (55) or the redistribution of NPY-Nanoluc to the constitutive pathway. However, together these nonspecific effects amount to less than half percent of all NPY-Nanoluc expressed in neurons. The fact that Rab3 deficient neurons do not show the same reduction in basal NPY-Nanoluc release as STXBP1-deficient neurons might be explained by a slightly increased mistargeting of NPY-Nanoluc in the absence of Rab3, a slightly increased susceptibility to cell rupture or the selective compensation by other factors during basal fusion events.

A previous report using a single-cell assay suggested that Dyngo-4a inhibits DCV exocytosis (56). In our assay, we did not observe this using NPY-Nanoluc (Fig. 2*C*). We confirmed the effectiveness of Dyngo-4a toward dynamin using a synaptophysin-pHluorin assay (Fig. S3*G*). Dyngo-4a is known to affect also dynamin-independent processes like fluid-phase endocytosis and peripheral membrane ruffling (57). It is possible that such effects lead to increased NPY-Nanoluc release, which mask the dynamin-dependent inhibition observed in single-cell assays. The dynamin-independent effect of Dyngo-4a might also be the cause of the observed increase in basal DCV fusion upon incubation with Dyngo-4a incubation.

The significant increase in basal release of NPY-Nanoluc was also detected when extracellular calcium was removed (Fig. 1*G*). DCVs exocytosis depends largely on extracellular calcium (42, 43, 58) and possibly directly or indirectly on calcium release from ER stores, regulated by the interplay between calcium sensors and calcium pumps located at ER and plasma membrane (59). Removal of extracellular calcium may affect calcium release from ER, which may again directly or indirectly influence basal DCV exocytosis.

Here, we compared the NPY-Nanoluc to NPY-pHluorin assay, a commonly used assay to study DCV exocytosis. NPY-pHluorin assays offer spatial resolution and in practice also a higher temporal resolution. However, neuronal diversity produces large cell-to-cell variation in such single-cell approaches which limits its throughput and makes it hard to produce robust, generalizable conclusions, especially when effects are partial, without excessive time investment (19, 20, 21, 22, 23, 30, 37). The sensitivity of NPY-Nanoluc assay, defined as the ability to detect statistically significant differences between two experimental groups for a given number of independent observations, was higher than the NPY-pHluorin assay (Fig. 3*A*). New single-cell methodologies, like single-cell RNAseq, have revealed a large diversity in neuronal cell types, which may also lead to substantial diversity in DCV exocytosis. For single-cell DCV exocytosis assays, it is difficult to avoid undersampling this diversity. Indeed, the coefficient of variation of evoked DCV exocytosis measured with NPY-pHluorin was 3.75 to 7.5 times higher than when measured with NPY-Nanoluc (Fig. 3*B* and Table 2). Hence, population-based assays, like the assay introduced here, are a solution to this problem, accounting for the full biological diversity in its parameters with reasonable efforts.

## Experimental procedures

### Primary mouse culture

All animals’ experiments were approved by institutional and Dutch Animal Ethical Committee regulations (DEC-FGA 11-03). Mouse pups of unknown sex were humanely sacrificed at embryonic day 18 by decapitation. Dissected hippocampi and cortexes were digested in Hank’s solution (Sigma) supplemented with 10 mM Hepes (Life Technology) and 0.25% trypsin (Life Technology) for 20 min at 37 °C. Next, the digested tissues were washed twice with Hank’s + 10 mM Hepes solution and once with Dulbecco's modified Eagle's medium, followed by trituration with fire polished glass Pasteur pipette. Neurons were plated on previously prepared coated plates in Neurobasal supplemented with 2% B-27, 18 mM Hepes, 0.25% GlutaMAX, 0.1% penicillin/streptomycin (P/S, Life Technology) at a density of 60,000 neurons per well on poly-L-Ornithine (Sigma)/laminin (Sigma) coated 24-well plate or 20,000 neurons on 96-well with previously prepared rat glia layer. Coated plates were obtained by incubation with solution containing poly-L-Ornithine (5 μg/ml end concentration) and laminin (2.5 μg/ml end concentration) for 2 h at 37 °C or 24 h at room temperature (RT).

### iPSC cell culture

iPSC line BIONi10-C-13, with NGN2 cassette that expression enforce differentiation into neurons, were purchased from Bioneer. iPSCs were maintained on Geltrex coated plates in essential 8 (Gibco) media supplemented with 0.1% P/S (Life Technologies).

### CRISPR KO line generation

The STXBP1−/− iPSC line was generated by CRISPR-Cas9–mediated gene engineering in BIONi10-C-13 with the guide RNA: TCATGGAGCACAGGGGAGCT, which targets c.721(T). The obtained STXBP1−/− line (SS241_C11 (KO-10)) was quality controlled by sequencing. The isogenic iPSC control line used in quality control and Western blot analysis of STXBP1 protein level underwent the same CRISPR-Cas9 procedure as SS241_C11 (KO-10) line.

### iPSC-derived human neuronal culture

iPSC differentiation toward neuronal fate was driven by Tet-inducible expression of NGN2 and supplementation with dual SMAD molecules. In short, on the first day of induction iPSCs were replated in N2 media (Dulbecco's modified Eagle's medium/F12 (Life Technologies) supplemented with 200 mM GlutaMAX (Life Technologies), 20% dextrose (Life Technologies), 1% N2 supplement B (StemCell Technologies), 0.1% P/S (Life Technologies)) to which dual SMAD inhibitors were added (100 μl LDN-193189 (Stemgent), 10 μM SB431542 (Torcis) and 2 μM XAV939 (Stemgent)) with Rock inhibitor (TetuBio), and 2ugl/ml doxycycline hyclate (Sigma). The following day culture media were refreshed to N2 prepared as during first day, with exception of Rock inhibitor, which was omitted. On the third day, culture media were replaced to N2 media supplemented with 10 μM FUDR (Sigma), dual SMAD inhibitors, and doxycycline hyclate (Sigma). On the fourth day, induced iPSCs were replated with accutase and plated on poly-L-Ornithine (Sigma)/laminin (Sigma) 24-well plate at density 50,000 neurons/well or on 96-well plate with previously prepared rat glia layer in neuronal culture medium (Neurobasal medium (Life Technologies) supplemented with: 200 mM GlutaMAX (Life Technologies), 20% dextrose (Life Technologies), nonessential amino acids (Life Technologies), B27 (Life Technologies), 0.1% P/S, 0.5% fetal bovine serum (Life Technologies)), supplemented with 10 ng/ml BDNF (Peprotech), 10 ng/ml ciliary neurotrophic factor (Stem Cell Technologies), and 10 ng/ml glial cell line-derived neurotrophic factor (Peprotech).

### Constructs and viral infection

The expression of luciferase reporter NPY-HA-Nanoluc or sec-Nanoluc was obtained by infection of neuronal culture with viral particles 6 to 7 days prior to the experiment, with the exception of cultures in Figure 5, where the iPSCs were infected with the viruses prior to the induction and differentiation into neurons. The viral particles were obtained by overexpression of pSyn-NPY-HA-Nanoluc-pLenti6.3 or pSyn-sec-Nanoluc-pLenti.6.3 in HEK293T cells, according to the protocol previously publish (60). pSyn-NPY-HA-Nanoluc-pLenti6.3 was obtained by fusion of HA-Nanoluc from pCMV-CD63-HA-Nanoluc to NPY and subcloning into pLenti6.3 backbone under Synapsin promoter. pSyn-sec-Nanoluc-pLenti6.3 was obtained by subcloning sec-Nanoluc from pCMV-sec-Nanoluc into pLenti6.3 under Synapsin promoter. pCMV-sec-Nanoluc and pCMV-CD63-HA-Nanoluc was obtained as a gift from Prof. Michiel Petgel.

### Luciferase reporter

Neurons were plated at high density in poly-L-Ornithin/laminin coated 24-well (50–60 k/well) or 96-well (15–25 k/well) with previously prepared glia layer. Neuronal cultures were washed 3x with Tyrode’s solution [119 mM NaCl, 2.5 mM KCl, 2 mM CaCl2∗2H2O, 2 mM MgCl2∗6H2O, 25 mM Hepes, and 30 mM glucose∗H2O, pH 7.4, mOsmol 280]. The basal and evoked release of NPY-Nanoluc was measured by incubation with Tyrode’s solution for 5 min, immediately followed by 5 min incubation with Tyrode’s solution with 60 mM KCl [61.5 mM NaCl, 60 mM KCl, 2 mM MgCl2∗6H2O, 2 mM CaCl2∗2H2O, 25 mM Hepes, and 30 mM glucose∗H2O, pH 7.4, mOsmol 280]. Media from both stimulations were then collected and precleaned by spinning down for 5 min at 200×*g* to remove disattached cells. The amount of NPY-Nanoluc reporter in precleaned media was obtained by adding Nano-Glo Luciferase Assay Reagent (Promega) and measuring luminesce RLU with luminesce plate reader. Neuronal cultures after double stimulation were then lysed with Nano-Glo Luciferase Assay Reagent (Promega), the luminesce signal measured from the lysates were used to normalize release of the reporter to the cell amount for each well.

For the experiment in Figure 1*G* media were adjusted to remove extracellular calcium by replacing 2 mM CaCl2∗2H2O with additional 2 mM MgCl2∗6H2O and adding 5 mM EGTA; adjusted evoked release’s buffer: [61.5 mM NaCl, 60 mM KCl, 4 mM MgCl2∗6H2O, 5 mM EGTA, 25 mM Hepes, and 30 mM glucose∗H2O, pH 7.4, mOsmol 280] and basal release’s buffer: [119 mM NaCl, 2.5 mM KCl, 4 mM MgCl2∗6H2O, 5 mM EGTA, 25 mM Hepes, and 30 mM glucose∗H2O, pH 7.4, mOsmol 280].

In the experiment in Fig. S2 different concentrations of extracellular calcium were obtained by adding appropriate amount of CaCl2∗2H2O to previously prepared basal release’s buffer: [ 119 mM NaCl, 2.5 mM KCl, 2 mM MgCl2∗6H2O, 25 mM Hepes, and 30 mM glucose∗H2O, pH 7.4, mOsmol 280] and evoked release’s buffer: [61.5 mM NaCl, 60 mM KCl, 2 mM MgCl2∗6H2O, 25 mM Hepes, and 30 mM glucose∗H2O, pH 7.4, mOsmol 280].

For the experiment in Figure 5 and Fig. S6, *D* and *E*, culture media were refreshed to culture media supplemented with either TTX or DMSO for either 2 h, 6 h, or 24 h. Then culture media were collected and spun down twice (first time at 200x, second time at 2000*g*), and the luminescence was measured from both precleaned media and corresponding cells lysates.

### Immunostaining

Neurons plated on previously etched and poly-L-Ornithine/laminin–coated glass coverslips were fixed by incubation with 3.7% formaldehyde (Merc) for 20 min. Then coverslips were washed twice with PBS, permeabilized with 0.5% Triton X-100 (Thermo Fisher Scientific) for 5 min and incubated with blocking solution [2% normal goat serum (Thermo Fisher Scientific), 0.1% Triton X-100 (Thermo Fisher Scientific) in PBS] for 1 h. After blocking, samples were incubated with primary antibodies diluted in blocking solution for overnight at RT. Used primary antibodies are as follows: chicken anit-MAP2 (Abcam; 1:500), rabbit anti-HA (Cell Signaling, 1:500), mouse anti-HA (Covance, 1:200), rabbit anti-NPY (Cell Signaling, 1:400), and rabbit anti-ChrA (SySy,1:300). Next, cultures were washed 3x with PBS and incubated with secondary antibody Alexa Fluor (Invitrogen, 1:1000) diluted in blocking solution for 2 h at RT. Later, fixed and stained coverslips were washed again 3x with PBS and mounted in Mowiol-DABCO.

### Western blot analysis

Cell lysates were prepared from WT and STXBP1−/− iNeurons cultured on poly-L-Ornithine/laminin plates by washing twice with PBS and scraping with PBS containing 100x protease inhibitor E-64d (Sigma, E8640). Collected cells were then spun down for 5 min at 12,000 rpm and lysated in Laemmli sample buffer containing 2% SDS (VWR chemicals, M107), 10% glycerol (Merck, 818709), 0.26 M β-mercaptoethanol (Sigma, M3148), 60 mM Tris–HCl (Serva, 37180) pH 6.8, and 0.01% bromophenol blue (Applichem, A3640). Sample were boiled at 100 °C for 5 min, separated on 10% SDS-polyacrylamide gels with 2,2,2-trichloroethanol and then transferred to nitrocellulose membrane 0.2 μm (Bio-Rad #1620112). The membrane was then blocked with 2% protease-free bovine serum albumin (268131000, ACROS Organics) in PBS-0.05% Tween solution for 1 h at RT and incubated with primary antibodies anti-Munc18 (610336, BD Transduction Laboratories, 1:5000) or anti-tubulin (T5326, Sigma-Aldrich, 1:1000) at 4 °C overnight. Subsequently the membrane was washed with PBS-0.05% Tween three times and incubated with secondary antibodies IRDye 680LT goat anti-mouse igg secondary antibody (926-68020, LI-COR) for 30 min at RT. Samples on the membrane were visualized with LI-COR Odyssey Fc, the quantification of band intensity was performed in Fiji ImageJ (https://fiji.sc/).

### Synaptophysin-pHluorin analysis

Hippocampal neurons were infected with synaptophysin-pHluorin 6 days prior the experiment. Neurons at DIV16 to 17 were placed in the imaging chamber and perfused with Tyrode’s solution at RT. Each neuron underwent the same stimulation protocol consisting of: 30 s baseline, 5s of 20 Hz electrical field stimulation (delivering 1 ms 30 mA pulses), 5 s perfusion Tyrode’s solution containing NH_4_Cl, 5 min treatment with Dyngo-4a, 5s of 20 Hz electrical field stimulation, and 5 s of perfusion with Tyrode’s solution at pH = 5.5. Images were acquired on an Eclipse Ti2 with a λ 60x oil objective (N.A. = 1.35) using NIS-Element 5.30 software (https://www.nikon.com) on a Prime-52B EM-CCD camera, using 470 nm LED illumination at 2 Hz. Images were analyzed in Fiji ImageJ (https://fiji.sc/).

### Power analysis

The effect size (Cohen’s d) was described as mean differences between two groups divided by batch corrected SDs ($Cohen^{\prime }s\;d=\frac{M1-M2}{\text{SD}}$). The SDs for each sample set were first batch corrected by using mean-centered method as described before (61), and then SD used in Cohen’s d was calculated with following formula: $=\frac{\sqrt{{SD1}^{2}-{SD2}^{2}}}{2}$ , where SD1 and SD2 stands for SD of sample set 1 and 2, respectively. The generation of power curved and prediction of sample size per group needed for 80% power was calculated with python package *statsmodels.stats.power* and *TTestIndPower*.

## Data availability

All materials and data are available upon reasonable request to the corresponding authors.

## Supporting information

This article contains supporting information.

## Conflict of interest

The authors declare that they have no conflict of interest with the contents of this article.
