# Supplementary material for: High-throughput assay for regulated secretion of neuropeptides in mouse and human neurons
Source: J Biol Chem. 2024 Apr 25;300(6):107321. doi: 10.1016/j.jbc.2024.107321 (PMC11170154; doi:10.1016/j.jbc.2024.107321)
Supplement: Supporting Information [file mmc1.docx]

**Supplementary Figures Legends**

**Supplementary Figure 1. NPY-Nanoluc reports DCV exocytosis in rodent CNS neurons.**

(A) Evoked/Basal ratio of released fractions upon stimulation of hippocampal neurons for 2 or 5 min with 2.5 mM KCl or 60 mM KCl.

(B) Bar plot of RLU signal from cells lysates of Rab3QKO and Rab3TKO hippocampal neurons.

(C) Evoked/Basal ratio of released fractions upon stimulation of Rab3QKO and Rab3TKO neurons.

(D) Bar plot of RLU signal from cells lysates of hippocampal neurons stimulated in 2mM or 0mM extracellular calcium.

(E) Evoked/Basal ratio of released fractions upon stimulation in 2mM or 0mM extracellular calcium.

For nonnormally distributed data: bar represents median value with 95% confidence interval, single dot represents measurement from single well. Mann-Whitney was used. *p<0.05, **p<0.01, ***p<0.001, ns p>0.05.

For normally distributed data: bar represents mean value with SD, single dot represents measurement from single well. Unpaired t-test was used, *p<0.05, **p<0.01, ***p<0.001, ns p>0.05. Table 1 shows results of statistical tests.

**Supplementary Figure 2. NPY-Nanoluc reports increased DCV exocytosis with increasing extracellular calcium.**

(A) Bar plot of released fraction upon 5min stimulations of cortical neurons in different extracellular calcium concentrations (0mM – 5mM). The concentration of calcium was adjusted for both evoked (60mM KCl) and basal (2.5mM KCl) release buffers.

(B) Evoked/Basal ratio of released fractions upon stimulation in different concentrations of extracellular calcium (0mM – 5mM).

(C) Bar plot of RLU signal from cells lysates of cortical neurons stimulated in different extracellular calcium concentrations (0mM – 5mM).

Bar represents median value with 95% confidence interval, single dot represents measurement from single well, the highlighted, connected dots represent median value for each independent experiment. Kruskal-Wallis with Dunn’s post-hoc comparison used for comparison between different conditions, for comparison of basal:evoked release within the same condition Multiple Mann-Whitney was used. *p<0.05, **p<0.01, ***p<0.001, ns p>0.05. Table 1 shows results of statistical tests.

**Supplementary Figure 3. NPY-Nanoluc reports DCV exocytosis upon treatment with network activity modulators.**

(A) Evoked/Basal ratio of released fractions upon stimulation for hippocampal neurons treated with PMA (1uM) or DMSO. PMA was added only to the evoked stimulation buffer (60mM KCl).

(B) Bar plot of RLU signal from cells lysates for conditions presented in (A).

(C) Evoked/Basal ratio of released fractions upon stimulation for cortical neurons treated with Nimodipine (30uM) or DMSO. Nimodipine was added to both basal stimulation buffer (2.5mM KCl) and evoked stimulation buffer (60mM KCl)

(D) Bar plot of RLU signal from cells lysates for conditions presented in (C).

(E) Evoked/Basal ratio of released fractions upon stimulation for cortical neurons treated with Dyngo-4a (10uM) or DMSO. Dyngo-4a was added to both basal stimulation buffer (2.5mM KCl) and evoked stimulation buffer (60mM KCl).

(F) Bar plot of RLU signal from cells lysates for conditions presented in (E).

(G) Average signal from active synapses visualized with Synaptophysin-pHuorin, normalized as ΔF/F0 from 4 neurons before and after treatment with Dyngo-4a (10uM) for 5min.

For nonnormally distributed data: bar represents median value with 95% confidence interval, single dot represents measurement from single well. Mann-Whitney was used. *p<0.05, **p<0.01, ***p<0.001, ns p>0.05. Table 1 shows results of statistical tests.

For normally distributed data: bar represents mean value with SD, single dot represents measurement from single well. Unpaired t-test was used, *p<0.05, **p<0.01, ***p<0.001, ns p>0.05. Table 1 shows results of statistical tests.

**Supplementary Figure 4. NPY-Nanoluc as a high-throughput assay to study DCV exocytosis in iPSC-derived human neurons.**

(A) Bar plot of Western Blot quantification of STXBP1 protein levels in WT and STXBP1-/- iNeurons at week 5-6, normalized to the tubulin levels. On the right: typical example of the blot stained with STXBP1 and Tubulin antibodies. Bar represents mean value with SD, single dot represents mean value for each independent experiment.

(B) Evoked/Basal ratio of released fractions for iNeurons stimulated in 0mM extracellular calcium (the concentration of calcium was adjusted for evoked release buffer), and homozygous STXBP1 null mutant in iNeurons at DIV14 in 24-well plate without glia.

(C) Bar plot of RLU signal from cells lysates for conditions presented in (B).

(D) Evoked/Basal ratio of released fractions upon stimulation for iNeurons (DIV21-25) treated with 4-AP (100nM), CNQX (10uM), PMA (1uM), or DMSO. All three compounds were added to washing buffer, basal and evoked release buffers. As a control for evoked release in 0 Ca2+ condition, neurons were treated with normal basal release buffer (2mM Ca2+, 2.5mM KCl).

(E) Evoked/Basal ratio of released fractions upon stimulation for iNeurons (DIV21-25) treated with BFA (5uM) for either 5min, 2h, 6h, or 10h. BFA was added to washing buffer, basal and evoked release buffers.

For nonnormally distributed data: bar represents median value with 95% confidence interval, single dot represents measurement from single well (for data presented in B-E). Kruskal-Wallis with Dunn’s post hoc comparison was used. *p<0.05, **p<0.01, ***p<0.001, ns p>0.05. Table 1 shows results of statistical tests.

**Supplementary Figure 5. Nanoluc assay for the detection of other secretory pathways in iNeurons.**

(A) Fold change (DMSO/TTX) of released fraction of NPY-Nanoluc (blue) or sec-Nanoluc (green) at different incubation time presented in Figure 5 A,C.

(B) Bar plot of released fraction of NPY-Nanoluc (NPY) or NLS-Nanoluc (NLS) in culture media over different incubation time (2h, 6h, 24h) with either DMSO or TTX (1uM) in iNeurons (DIV21-25).

(C) RLU signal from cells lysates expressing NPY-Nanoluc (NPY) or NLS-Nanoluc (NLS) for the conditions presented in (B), normalized to the DMSO control.

For nonnormally distributed data: bar represents median value with 95% confidence interval, single dot represents measurement from single well. Multiple Mann-Whitney was used. *p<0.05, **p<0.01, ***p<0.001, ns p>0.05. Table 1 shows results of statistical tests.

**Supplementary Figure 6. NPY-Nanoluc detects changes in basal DCV exocytosis in rodent CNS neurons.**

(A) Bar plot of NPY-Nanoluc basal release (2.5mM KCl) in hippocampal neurons (DIV15) upon treatment with DMSO or AP5(50uM)/DNQX(10uM). The inhibitors (AP5/DNQX) were added to washing buffer, basal and evoked release buffers.

(B) Bar plot of evoked NPY-Nanoluc release for the condition presented in (A).

(C) RLU signal from cell lysates for conditions presented in (A-B).

(D) Bar plot of basal NPY-Nanoluc release in culture media over 2h with DMSO, TTX (1uM), AP5 (50uM)/DNQX(10uM) or non-treated control (CTR), in hippocampal mouse neurons cultured in 96-well plate with glia feeder layer.

(E) RLU signal from cells lysates expressing NPY-Nanoluc for the conditions presented in (D).

For normally distributed data: bar represents mean value with SD, single dot represents measurement from single well. Ordinary one-way ANOVA comparison was used. *p<0.05, **p<0.01, ***p<0.001.

For nonnormally distributed data: bar represents median value with 95% confidence interval, single dot represents measurement from single well. Kruskal-Wallis or Mann-Whitney comparison was used. *p<0.05, **p<0.01, ***p<0.001. Table 1 shows results of statistical tests.
