# Supplementary material for: High-throughput assay for regulated secretion of neuropeptides in mouse and human neurons
Source: J Biol Chem. 2024 Apr 25;300(6):107321. doi: 10.1016/j.jbc.2024.107321 (PMC11170154; doi:10.1016/j.jbc.2024.107321)

Supplementary Figure 2. NPY-Nanoluc reports increased DCV exocytosis with increasing extracellular calcium.

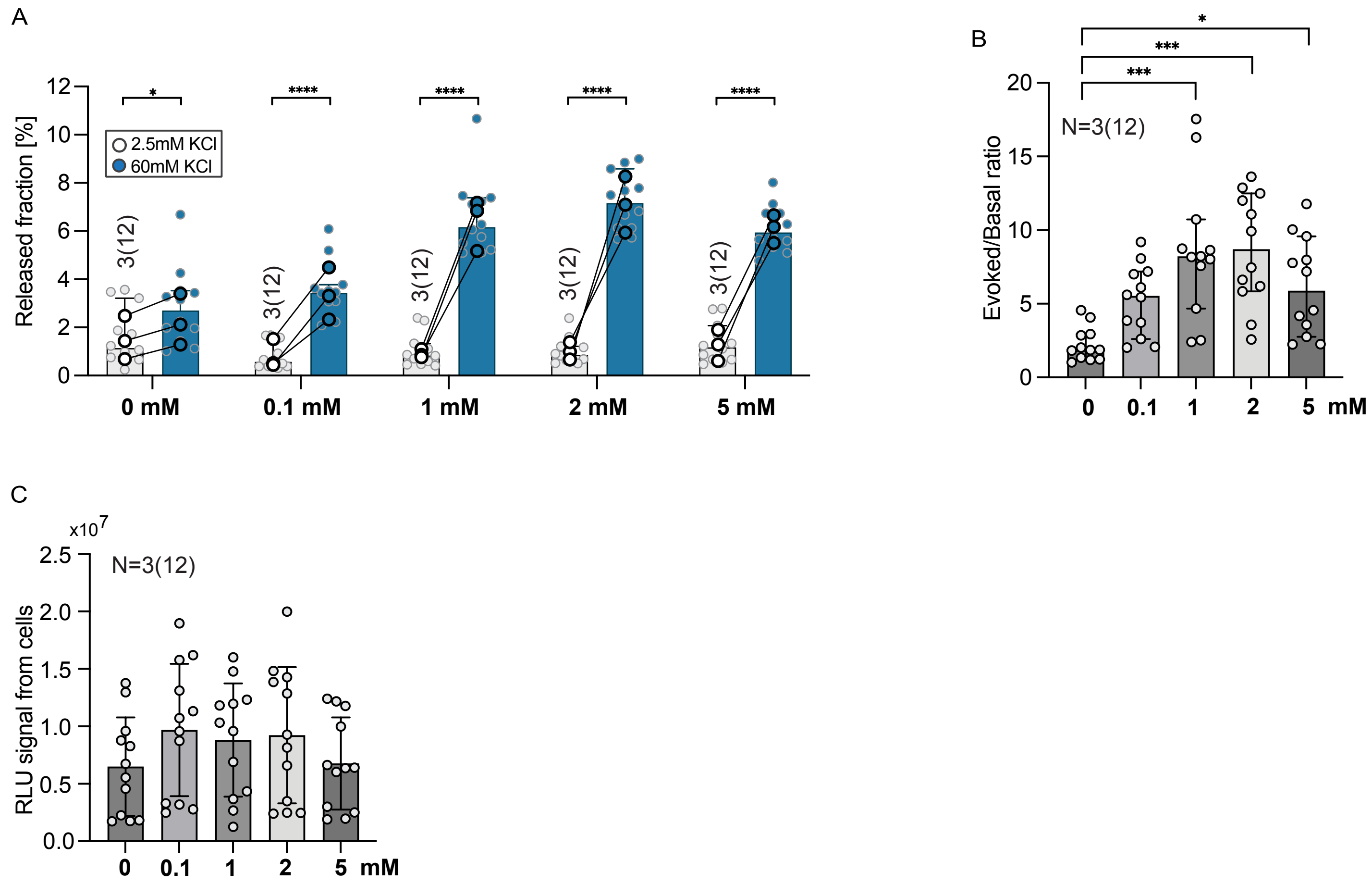

Supplement: Supporting Figure S2 [file mmc3.pdf]
