# Supplementary material for: High-throughput assay for regulated secretion of neuropeptides in mouse and human neurons
Source: J Biol Chem. 2024 Apr 25;300(6):107321. doi: 10.1016/j.jbc.2024.107321 (PMC11170154; doi:10.1016/j.jbc.2024.107321)

Supplementary Figure 3. NPY-Nanoluc reports DCV exocytosis upon treatment with network activity modulators.

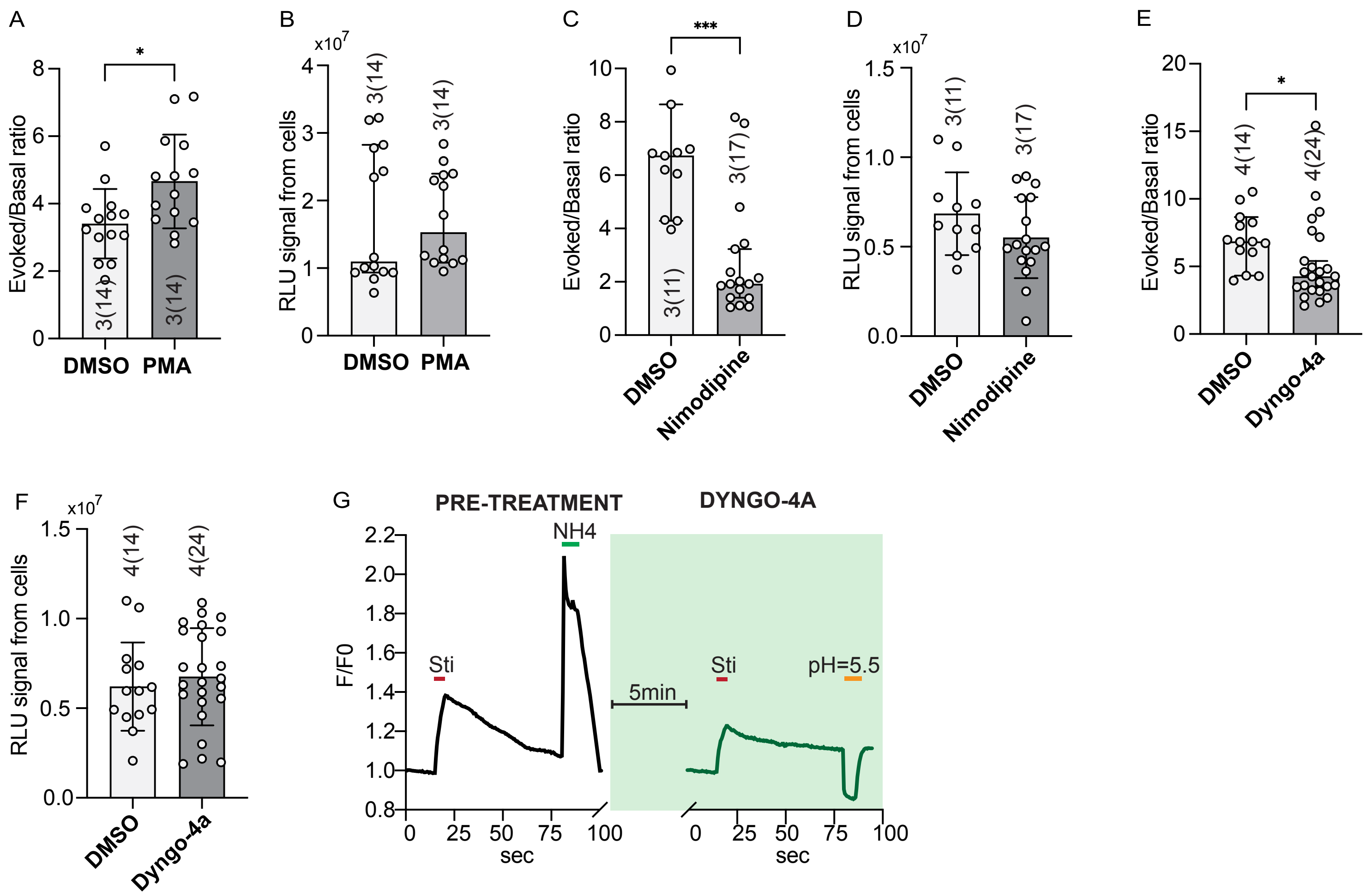

Supplement: Supporting Figure S3 [file mmc4.pdf]
